# Temporal differences and commonalities between hand and tool neural processing

**DOI:** 10.1038/s41598-023-48180-8

**Published:** 2023-12-14

**Authors:** L. Amaral, G. Besson, E. Caparelli-Dáquer, F. Bergström, J. Almeida

**Affiliations:** 1 Proaction Laboratory, Faculty of Psychology and Educational Sciences, University of Coimbra, Coimbra, Portugal; 2 Department of Neuroscience, Georgetown University Medical Center, Washington, DC USA; 3 CINEICC, Faculty of Psychology and Educational Sciences, University of Coimbra, Coimbra, Portugal; 4 Laboratory of Electrical Stimulation of the Nervous System (LabEEL), Rio de Janeiro State University, Rio de Janeiro, Brazil; 5 Department of Psychology, University of Gothenburg, Gothenburg, Sweden

**Keywords:** Cognitive neuroscience, Neural decoding

## Abstract

Object recognition is a complex cognitive process that relies on how the brain organizes object-related information. While spatial principles have been extensively studied, less studied temporal dynamics may also offer valuable insights into this process, particularly when neural processing overlaps for different categories, as it is the case of the categories of hands and tools. Here we focus on the differences and/or similarities between the time-courses of hand and tool processing under electroencephalography (EEG). Using multivariate pattern analysis, we compared, for different time points, classification accuracy for images of hands or tools when compared to images of animals. We show that for particular time intervals (~ 136–156 ms and ~ 252–328 ms), classification accuracy for hands and for tools differs. Furthermore, we show that classifiers trained to differentiate between tools and animals generalize their learning to classification of hand stimuli between ~ 260–320 ms and ~ 376–500 ms after stimulus onset. Classifiers trained to distinguish between hands and animals, on the other hand, were able to extend their learning to the classification of tools at ~ 150 ms. These findings suggest variations in semantic features and domain-specific differences between the two categories, with later-stage similarities potentially related to shared action processing for hands and tools.

Our ability to recognize objects is crucial in our daily life in order to guide and adapt our behavior to our needs and the context in which we are in. Research in object recognition has been trying to unravel the neural processes behind object recognition using different approaches ranging from neuropsychological to brain imaging techniques^1–4^. A central aspect in object recognition is understanding how object knowledge is organized in the human brain: i.e., understanding where and how object knowledge is stored and organized^5^. Importantly, knowing when different kinds of object-related information become available or differentially organized is also key for unravelling object recognition. Here, we will focus on the temporal dynamics of object knowledge.

Object recognition occurs in a fraction of a second, and it is a highly structured process. Several functional Magnetic Resonance Imaging (fMRI) studies have shown that specific categories of objects elicit higher responses (when compared to baseline categories) in different regions of the brain (e.g., faces^6^; places/scenes^7^; tools^8–10^; bodies^11^; and hands^12,13^). But what drives this categorical organization? Different theories try to explain this object topography by appealing to modality-specific effects^14,15^, domain-specific constraints^16,17^, or constraints imposed by connections with distal regions that share the same categorial preference^8,18–22^ (see also^17,23^), among others.

These studies have all focused on a static spatial understanding of object processing. Nevertheless, the temporal layout of object processing is essential for a more complete understanding of how we recognize objects in order to navigate our environment. In fact, electrophysiological studies have been trying to identify the time correlates of object processing^24–28^ (for a review see^29^). The existence of a hierarchical structure is a prominent characteristic shared by both visual processing and object recognition. Many studies have shown how object representations are temporally stratified going from a fast and coarse categorization to a slower and more detailed representation^30–33^. For instance, in a study combining fMRI and magnetoencephalography (MEG), Cichy and colleagues showed not only a temporally organized processing sequence underlying object recognition, but they also demonstrated that object representations are organized categorically^32^ (see also^31^). Using representational similarity analysis^34^, they fused fMRI and MEG signals and showed that early visual representations appear in the occipital lobe at around 50-80 ms after stimulus onset. This neuronal activity then expands in time and space into ventral and dorsal visual stream regions, showing a clear temporal pathway from low- to high-level visual processing. They also showed that the effects observed within the ventral stream were category-selective, supporting previous research on temporal dynamics^30,35^.

Obtaining a temporal layout of object processing is thus a promising avenue for understanding object-related computations, and it complements our spatial understanding of how objects are processed in the brain. Importantly, though, a temporal understanding of object processing can go beyond just temporally tagging our spatial understanding of object knowledge: in fact, it can help adjudicate different computational hypotheses about object-related neural processing.

This may be particularly important for regions that seem to show spatially defined overlapping categorical preferences for more than one category or objects. One such example is the spatial overlap in categorical preferences for hands and tools. Despite their perceptual differences, hands and tools show an overlap in neural response preferences in different cortical regions like the ventral premotor cortices, the left lateral occipitotemporal cortex (LOTC) and the left inferior parietal lobe (IPL)^12,36,37^. According to Bracci and colleagues^12^, this overlap cannot be explained by shared visual/perceptual features and reflects the common specific set of features that hands and tools share during object manipulation—i.e., they both relate to visuomotor and action processing. Moreover, we have recently shown that these two categories are, in fact, functionally related^38^. Through our study, we demonstrated that the unconscious processing of images from one category interferes with the recognition of images from the other category. Specifically, processing an image of a hand delays the subsequent processing of a tool image, and vice versa^38^. This functional relationship plays a major role in our daily behavior and how we interact with objects. In line with this idea is Gibson’s principle^39^ that an object automatically communicates certain action possibilities (i.e., affordances). Several electroencephalographic (EEG) studies have focused on tools and found that early components (e.g., N1, P1, N2) are modulated by action affordances and affordance congruency^40–45^, highlighting the visuomotor processing shared between hands and tools.

Can category-specific processing and computations about these two categories be disentangled from these overlap areas? In a previous fMRI study^18^, we investigated category-specific processing in the brain, specifically focusing on hand and tool representations. We found that despite the presence of particular tool-specific and hand-specific fMRI overlapping responses in left IPL and left LOTC, the connectivity patterns from these regions to the remaining brain were dependent on the category being processed. The study suggested that distinct neural networks exist for each category, even within overlapping regions. More recently, we showed that applying a neuromodulation technique (e.g., transcranial direct current stimulation—tDCS) to left LOTC, and combining it with a tool related task, improves the processing of tools distally in nodes of the tool network^46^. These findings suggest that despite the overlap observed in left LOTC, it is possible to disentangle functionally-specific networks by applying tDCS combined with a category-specific task.

Perhaps another way in which the putative spatial neural overlap is resolved is by looking at whether hand and tool computations are implemented in different time points—that is, perhaps the time-course of hand and tool processing may be different. Here we tested how temporally separate hand and tool processing are. We hypothesized that, despite their spatial overlap, distinct temporal patterns would emerge for hand and tool processing. To do so we used a multivariate approach where we compared classification accuracy (for the different time points of an object categorization task) for images of hands and for images of tools when compared to images of animals. The application of multivariate analyses to EEG data is rather recent, but it provides a robust methodology to access temporal neural dynamics^47^.

## Methods

### Participants

Fourteen participants (M = 28 years, SD = 8.01, 6 males) took part in this study. This sample size follows previously EEG studies that described group-level object category with a similar number of participants^27,33,44^. All participants had normal or corrected to normal vision and were right-handed. Written informed consent was obtained from all participants prior to the beginning of the experiment and the study was approved by the Ethical Committee of the Faculty of Psychology and Educational Sciences of the University of Coimbra. All experiments were carried out in accordance with relevant guidelines and regulations.

### Stimuli and procedure

**Figure 1:**
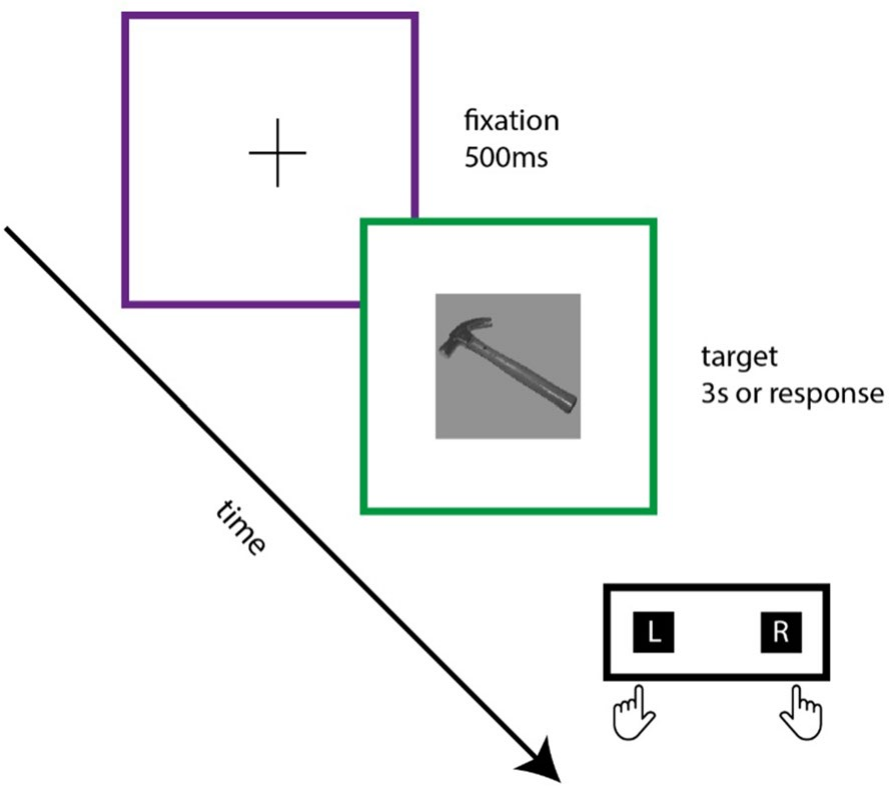
Experimental procedure. A fixation cross appeared for 500 ms followed by the target image. The task of the participants was to press a button for one category (hand or tool) or the other button for the control category (foot or animal).

Participants were asked to categorize images of hands, tools, animals and feet, following a paradigm that was partially used in a previous study^38^ (Fig. 1). In this prior study^38^, the use of animals and feet as controls for the tool and hand categories, respectively, was found to be efficient and adequate. The primary purpose of the current paradigm was to use animals and feet as control categories. However, as we describe below feet turned out to be an inappropriate control category because distinguishing between tools and feet is considerably easier than distinguishing between hands and feet. Furthermore, because animals have been extensively utilized as a control category in visual categorization research, we decided to focus the remaining analyses only on this control category.

**Figure 2:**
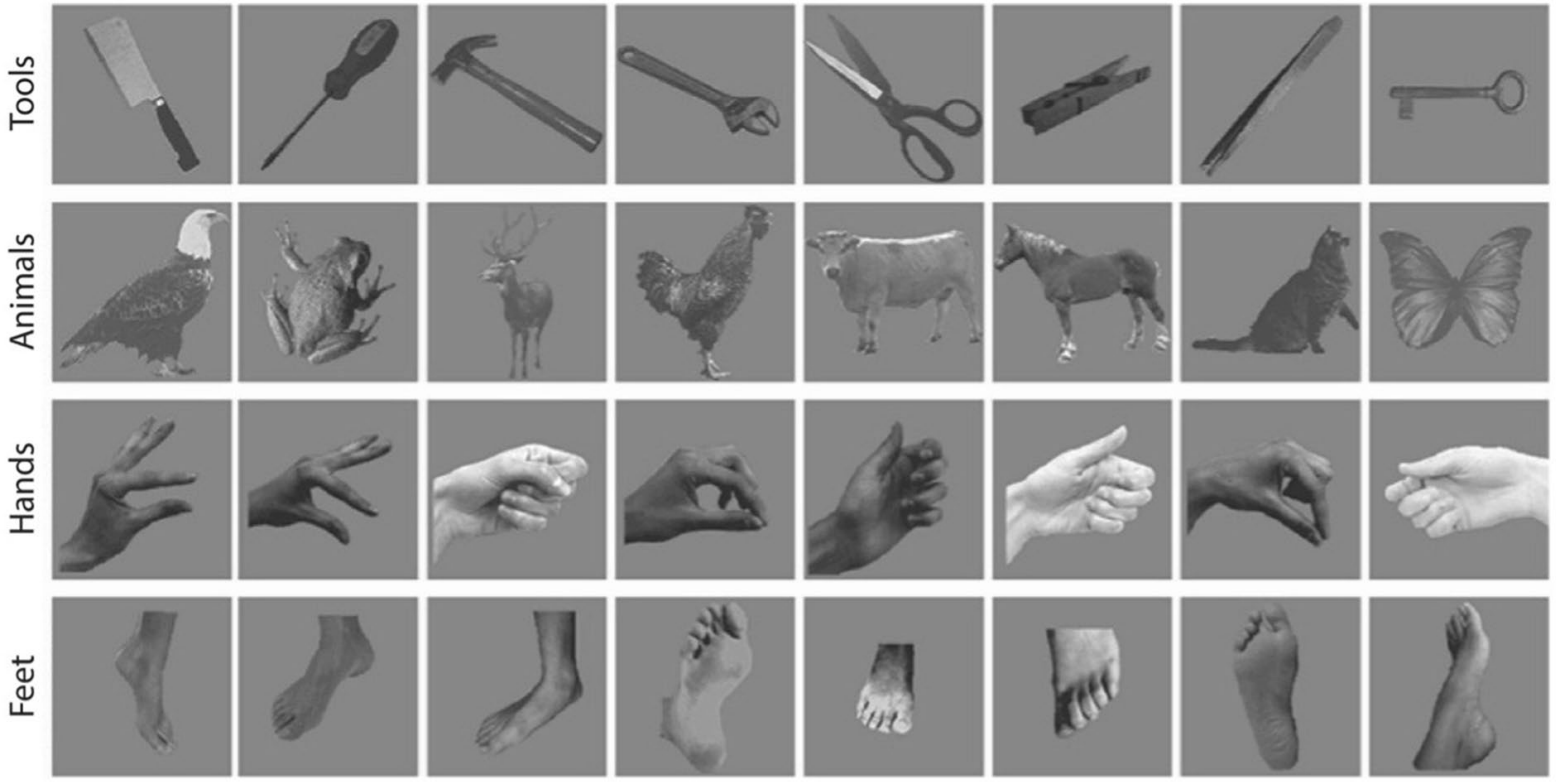
Stimuli used in the experiment. Here we present examples of the stimuli used in the experiment for each category.

Stimuli were greyscale, 200*200 pixels (subtending ~ 5° of the visual angle) and each category included 8 pictures of different objects/items (total = 32 pictures, Fig. 2). Tool pictures were photographs of different manipulable objects (tweezers, key, spring, scissors, wrench, hammer, screwdriver, and knife). Hand pictures were photographs of hands in grasp position, matching the type of grip of the tool images. There were no significant differences between the images in mean luminance and object size was qualitatively controlled to ensure that all items presented similar surface area. Stimuli were presented using Matlab (The MathWorks Inc., Natick, MA, USA) and Psychtoolbox^48^ using a refresh rate of 60 Hz.

After setting up the EEG device and placing the electrodes, participants started the task. On each trial, a fixation cross was presented during 500 ms, followed by the target picture (that stayed on screen for 3 s or until the participant responded). The instructions were to press a button, as quickly and accurately as possible, with their left or right index, indicating category membership of the picture that was presented. The experiment was divided in two parts: one part where participants categorized tools vs. animals; and another part where participants categorized hands vs. feet. Response assignment for the buttons was counterbalanced across participants, as well as the order of the two categorization tasks. There were 128 trials for each category/condition and the number of repetitions of each stimulus was 16, for a total of 512 trials.

### Data acquisition

Electrical brain activity was recorded using a wet-based elastic cap with 64 channels (eego™mylab, ANT Neuro, The Netherlands). Data was acquired with a sampling rate of 1000 Hz. The impedance of all electrodes was kept below 5 KΩ. EEG signal was recorded using EEProbe recording software (ANT Neuro, The Netherlands) and was amplified using an ANT digital amplifier.

### Data preprocessing

Preprocessing was performed in Matlab (The MathWorks Inc., Natick, MA, USA) using the open source EEGLAB toolbox^49^, and custom-made scripts. EEG data was down-sampled to 250 Hz, digitally filtered using a bidirectional linear filter (EEGLAB FIR filter) that preserves phase information (pass-band 0.5–40 Hz), and then re-referenced offline on the average of both mastoids. EEG data underwent a custom-made sanity check and correction to control that the EEG triggers (sent from the task computer and recorded by the amplifier in EEG data) correctly matched the task triggers (sent from and recorded by the task computer). Triggers and corresponding EEG trials were removed when no correction could be ensured (2 trials of one participant were lost). EEG data was then epoched (from  − 500 to 500 ms post-stimulus onset) and baseline-corrected (by subtracting the EEG average from the window  − 200 to 0 ms post-stimulus onset).

*EEG automatic artifact rejection.* An automatic artifact rejection algorithm^50^ was then used to reject bad electrodes and/or trials. Briefly, considering a group of time signals (e.g., epochs of trials along one electrode), this algorithm reject those presenting a peak-to-peak value (a quantity commonly used for identifying bad trials in M/EEG) exceeding a (data-driven) threshold, automatically defined as the threshold yielding the minimum difference (i.e., sum of the squared difference) between the mean of the under-threshold signals and the overall median signal^50^. For each participant, this algorithm was applied twice in the following order: 1) *across trials* for each electrode, in order to detect electrode-wise bad trials; and 2) *across electrodes* for each trial, in order to detect trial-wise bad electrodes. After each application of the algorithm, each trial was repaired by interpolation if less than half the electrodes were rejected or excluded from subsequent analysis otherwise. Overall, this two-steps procedure yielded a rejection rate of 8.10% (3.75%; mean and SD across participants) of the data (channels and/or trials); trial-wise electrode interpolation could then be applied to the extent that our final dataset missed 4.42% (3.31%) of the trials.

### Multivariate pattern analysis

Classification analyses were performed using the Matlab toolbox CoSMoMVPA^51^. At each time point ( − 100 to 500 ms post-stimulus onset), linear discriminant analysis (LDA) classifiers were used on z-score-normalized EEG signals of our experimental conditions. Two distinct classification approaches were conducted. In a first ‘typical classification’ approach, we focused on the decodability of four specific pairs of conditions: hands vs. animals, tools vs. animals, hands vs. feet, tools vs. feet. In a further ‘cross-classification’ approach, classifiers were trained to discriminate between hands vs. animals, and then tested on tools extracting the accuracy for classifying tools as hands or animals (and vice-versa, training on tools vs. animal and testing on hands). To investigate whether the neural information identified through cross-classification at time t persisted at time t’, we further used a temporal generalization (TG) method^52^ over the cross-classification, assessing how the classifiers could generalize across time points. Because the classification approach revealed baseline-dependent differences (see results for more information), we only used animals as the baseline category for the temporally generalized cross-classification analysis.

For each participant, the classification accuracy was computed across all electrodes (63 in total, excluding EOG), for each of the 150 time points of interest (between -100 and 500 ms). A leave-one-fold-out (k = 10 folds) cross-validation procedure was used to assess the performance of the classifiers to ensure that training and testing data was kept completely independent, and that partitions were balanced (i.e., each condition was presented equally in each fold/partition). Statistical significance was then determined at the group level using threshold-free cluster-enhanced^53^ (TFCE) and maximal statistic permutation testing^54^ to correct for multiple comparisons and obtain a z-score map across timepoints. Briefly, this consisted in comparing the TFCE-transformed original classification accuracy to a null-distribution (N = 100) of the maximal statistic over the timepoints of the TFCE-transformed classification accuracy (as implemented in the CoSMoMVPA toolbox^51^) that were obtained after randomly permuting labels of the conditions across trials 100 times. The assignment to training and testing folds in the cross-validation procedure of the original classification accuracy and the resulting statistical comparison to the null-distribution was repeated N = 20 times, resulting in 20 z-score maps for a given analysis, which we then averaged into a final one. Significance cutoffs for z-values were two-tailed and set to *p* < 0.05 (i.e. |z|> 1.96).

## Results

### Behavioral results

We used a one-way repeated measures ANOVA to analyze accuracy and reaction times across conditions. Accuracy results show that the experimental conditions did not differ in difficulty (*F*(1,13) = 1.07, *p* = 0.37). Reaction time (RTs) analysis also show that the four conditions did not differ in RTs (*F*(1,13) = 1.10, *p* = 0.36). For more details, please see Table 1.

**Table 1:** Behavioral results.

Condition	Accuracy		Reaction Times (in ms)	
	Average	SD	Average	SD
Hands	0.97	0.02	603	42.99
Tools	0.97	0.04	616	54.98
Feet	0.97	0.03	593	34.47
Animals	0.98	0.02	608	60.38

### Multivariate classification results

#### Are hands and tools differently processed over time?

To test whether and when are tools and hands differentially processed, we employed a multivariate approach and compared the classification accuracy between hands (vs. animals OR feet) and tools (vs. animals OR feet). Although our main comparison is between hands and tools, we opted against directly comparing hands and tools due to their distinct features, including visual characteristics and their existence in different domains. We expected that a classifier would effectively identify these two categories based on such attributes alone and demonstrated that this is actually the case (see Supplementary Fig. S1). Instead, our objective is to examine the instances where hands and tools differ, as well as when they exhibit similarities. To achieve this, we believe the most effective approach is to compare both hands and tools against to the same baseline.

**Figure 3:**
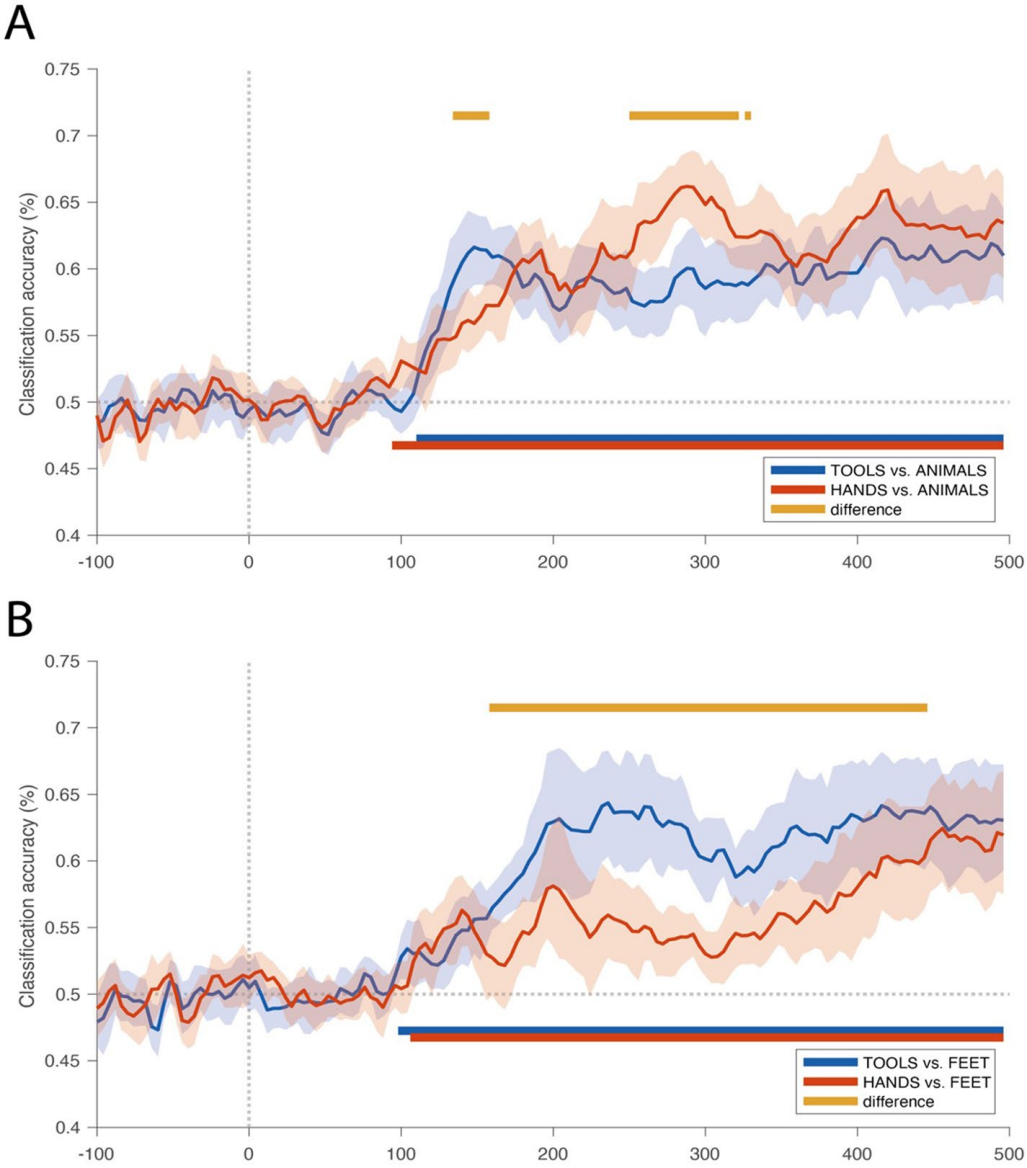
Classification accuracy results (paired analysis). (**A**) Classification accuracy for hands vs. animals (red line), and tools vs. animals (blue line). (**B**) Classification accuracy for hands vs. feet (red line), and tools vs. feet (blue line). The yellow points correspond to the significant time clusters (two-tailed, p < .05, i.e. |z*|*> 1.96), cluster-wise corrected for multiple comparisons using TFCE and maximal statistic permutation testing) for the paired analysis between hands and tools accuracy.

First, we analyzed each classification accuracy (acc) and their significance against chance. As shown in Fig. 3A, the discrimination between tools and animals (blue line) was significant starting at ~ 112 ms, whereas hands vs. animals (red line) started to become significant at ~ 96 ms (see also Table 2). The same applies to the discrimination between tools and feet (Fig. 3B, blue line) and between hands and feet (Fig. 3B, red line), with both being significant starting at ~ 100 ms and ~ 108 ms respectively (see also Table 2).

**Table 2:** Peak time points for the main categories (hands and tools), when compared to two different control categories (animals and feet).

	Early peak t-point	Mid peak t-point	Late peak t-point
Tools vs. animals	148 ms (z-value = 2.33, acc = .62)	288 ms (z-value = 2.33, acc = .60)	416 ms (z-value = 2.33, acc = .62)
Hands vs. animals	192 ms (z-value = 2.33, acc = .61)	288 ms (z-value = 2.33, acc = .66)	420 ms (z-value = 2.33, acc = .66)
Tools vs. feet	104 ms (z-value = 2.33, acc = .53)	236 ms (z-value = 2.33, acc = .64)	416 ms (z-value = 2.33, acc = .64)
Hands vs. feet	140 ms (z-value = 2.33, acc = .56)	200 ms (z-value = 2.33, acc = .58)	456 ms (z-value = 2.33, acc = .62)

We then compared the classification accuracy between hands vs. animals and tools vs. animals (see Fig. 3A, yellow line). Here, we showed that classification accuracy was significantly different between the two conditions during specific time ranges. The accuracy for tools (vs. animals) was significantly higher than hands (vs. animals) in an early time point between 136 and 156 ms (see also Table 3). The reverse outcome (i.e., accuracy for hand discriminations higher than the accuracy for tool discrimination) was observed in two later time intervals: between 252 and 320 ms and around 328 ms (see also Table 3). These results suggest important differences in the time-courses of hand and tool processing.

**Table 3:** Peak time points for the difference between the main categories (hands and tools).

	Early peak t-point	Mid peak t-point	Late peak t-point
[Tools vs. animals] VS [Hands vs. animals]	148 ms (z-value = − 2.33, acc_tools_ = .62, acc_hands_ = .56)	276 ms (z-value = 2.33, acc_tools_ = .58, acc_hands_ = .65)	328 ms (z-value = 2.06, acc_tools_ = .59, acc_hands_ = .65)
[Tools vs. feet] VS [Hands vs. feet]	172 ms (z-value = − 2.33, acc_tools_ = .58, acc_hands_ = .53)	264 ms (z-value = 2.33, acc_tools_ = .64, acc_hands_ = .55)	360 ms (z-value = − 2.33, acc_tools_ = .62, acc_hands_ = .55)

Nevertheless, a different pattern was obtained when using feet as the control baseline: accuracy for tools (vs. feet) was significantly higher than hands (vs. feet) over a wide time range from ~ 160 to ~ 444 ms, and the accuracy for hands (vs. feet) was never significantly higher than tools (see Fig. 3B, yellow line, see also Table 3). This fact could be easily explained by the fact that hands and feet are two very close categories (i.e., body parts). Because of this, we decided not to include the feet condition as a baseline in the following analyses because this baseline is not balanced for the two target categories (for the results with feet as the baseline category, please see Supplementary Fig. S2). Moreover, the use of the same baseline category (animals) for both classification procedures allows for a more balanced understanding of the differences in the processing of hands and tools across time.

#### Is the category-specific neural representation of tools/hands at different time points informative of the neural representation of hands/tools and when generalizing across time?

We then focus on whether category-specific information of tools (or hands) can be generalizable to the processing of hands (or tools). To address this question, we used a cross-classification approach, and tested 2 classifiers: (1) one trained on classification tools vs. animals; and (2) one trained on classification hands vs. animals. Importantly, we tested these classifiers with the other category of interest (e.g., if a classifier was trained on tools vs. animals, it was tested on hands). We also wanted to understand whether the generalization of tool/hand representations to the processing of hands/tools could be extended in time—that is, whether there was cross-time generalization of the processing of tools and hands. For this, we used a TG approach, and looked for timepoints where the classification ability for tools against animals could generalized to category of hands across time (and vice-versa). The results from this analysis are represented in a matrix, where each axis indicates the training and the testing time. Results that lie on the diagonal of the matrix represent the same time point for training and testing (i.e., a typical cross-classification analysis).

**Figure 4:**
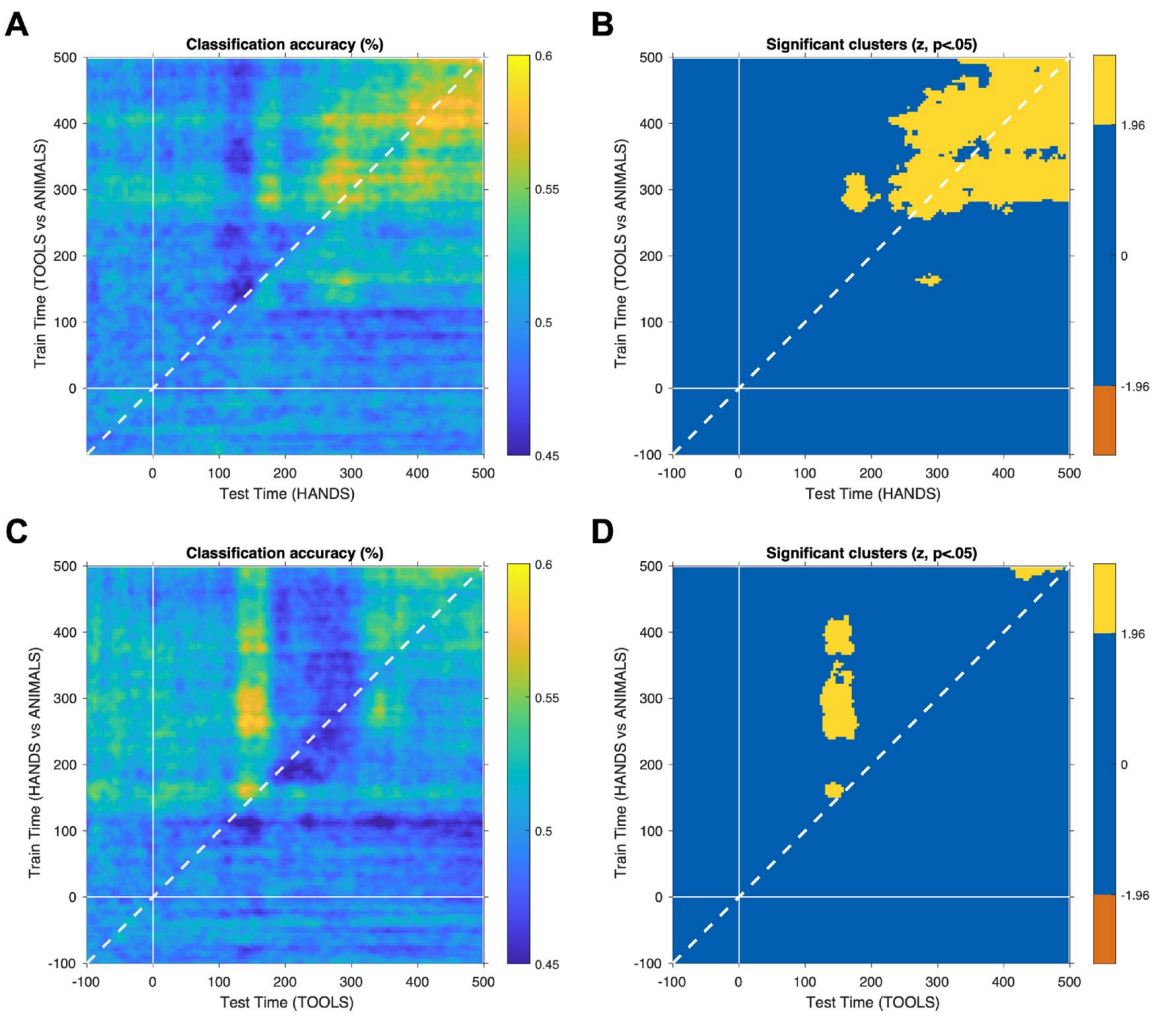
Results from time generalization approach using animals as the control category. (**A**) Classification accuracy across time when the classifier trained on tools vs. animals and was then tested on hands. (**B**) The yellow color represents the significant time points when classifying hands as tools (z > 1.96, cluster-wise corrected for multiple comparisons using TFCE and maximal statistic permutation testing). (**C**) Classification accuracy across time when the classifier trained on hands vs. animals and was then tested on tools. (**D**) The yellow color represents the significant time points when classifying tools as hands (z > 1.96, cluster-wise corrected for multiple comparisons using TFCE transform and maximal statistic permutation testing).

For the classifier that was trained on tools and tested on hands, we found significant generalization effects in two time windows between 260 and 320 ms and between 376 and 500 ms (peak: t-point = 288 ms, z-value = 2.33, acc = 0.57; see the diagonal on Fig. 4A,B). Moreover, when extending the analysis to different time points (that are arranged outside the diagonal), the results show that training on tools vs. animals (and testing on hands) revealed two major clusters (i.e., classifying hands as tools more than as animals; all clusters with a z > 1.96). The smaller cluster shows that a classifier trained on tools vs. animals with data from around 156–172 ms can be used to classify hands as tools in a later time interval (around 266–304 ms; Fig. 4A,B, see also Table 4). The big cluster shows that a classifier trained on tools vs. animals between ~ 284 and 500 ms classifies hands as tools both earlier (from ~ 156 ms) and later (until ~ 500 ms) in time (Fig. 4A,B, see also Table 4).

**Table 4:** Peak time points for the Time Generalization (TG) Effects.

	Peak t-point
Train on tools (vs. animals) test on hands	Smaller cluster: t-point_train_ = 160 ms, t-point_test_ = 292 ms (z-value = 2.22, acc = .57) Big cluster (early time window): t-point_train_ = 288 ms, t-point_test_ = 176 ms (z-value = 2.22, acc = .57) Big cluster (later time window): t-point_train_ = 400 ms, t-point_test_ = 416 ms (z-value = 2.33, acc = .59)
Train on hands (vs. animals) test on tools	Early time window: t-point_train_ = 164 ms, t-point_test_ = 140 ms (z-value = 2.33, acc = .58) Later time window: t-point_train_ = 260 ms, t-point_test_ = 140 ms (z-value = 2.33, acc = .59)

For the classifier trained on hands and tested on tools, we found a small generalization effect at ~ 152 ms (peak: t-point = 152 ms, z-value = 2.00, acc = 0.55; see the diagonal on Fig. 4C,D). Nevertheless, the TG results have extended this finding, showing that the classifier is able to decode tools at ~ 152 ms when it was trained not only at the same time (~ 152 ms) but also later between 240 and 424 ms (Fig. 4C,D, see also Table 4). The classification is again significant only later around 412 and 500 ms, when it was trained at ~ 480–500 ms (all clusters with a z > 1.96, Fig. 4C,D; see also Table 4).

## Discussion

Understanding the temporal dynamics of processing object knowledge is essential for developing sophisticated models of visual object recognition. Here we set out to investigate the timing of object knowledge by looking at two functional related categories: hands and tools. Specifically, we investigated whether there are temporal differences and/or similarities during the processing of these two related categories.

We first looked at whether neural patterns for tools and hands can be temporally discriminated. To this end, we used a classification approach, where we compared the accuracy of classification between hands and animals and between tools and animals during different time points after stimulus onset. Even though the classification accuracy profiles for these two classification conditions present some similarities through time, the accuracy pattern was clearly different during two time intervals (~ 136–156 ms and ~ 252–328 ms).

In the first time interval (~ 136–156 ms), the accuracy for classifying tools (vs. animals) was higher than that for classifying hands (vs. animals). These results show that at that time, tools are more different from animals than hands are from animals. This shows that during this earlier time interval, category-specific tool responses seem to be more distinguishable and/or unique than category-specific hand responses. This could be due to larger domain-specific differences (e.g., living vs. non-living distinctions^16^). For instance, it may be the case that domain membership (i.e., tools are non-living things, whereas animals and hands are living things) could be the driving force of the effect at this stage. In fact, in a MEG study, Carlson et al.^30^ used multidimensional scaling (MDS) and found that by ~ 120 ms after stimulus onset, distinguishability between exemplars becomes possible, and from that time point onwards we can see the emergence of categories and subcategories (e.g., at ~ 120 ms, human faces and animals are already quite distinct from man-made objects but still very close in the representational space, and from ~ 140 ms, they start to also distinguish one from another). These results can help us to explain why tools and animals are more distinguishable at an earlier stage than hands (a body part) and animals. Moreover, the fact that animals and man-made objects differ in early perceptual features^55^ and that categorization between these two categories emerges at ~ 150ms^27^ could potentially also be the basis of our results. Finally, tools and animals also immediately differ in their level of manipulability, a difference that is not immediately apparent when comparing hands and animals, which is another aspect that may lead to better classification between tools and animals than between hands and animals. In fact, Proverbio^56^ showed a desynchronization of the Mu (μ) rhythm around 140–175 ms, when comparing tools vs. non-tools (i.e., non-manipulable objects). The μ rhythm is a brain wave that appears most prominently over the sensorimotor cortex during a relaxed state and its suppression is induced by motor action. These results suggest then that motor information (e.g., manipulability) can be extracted from visual objects at an early stage of processing^56^. Finally, another aspect that could explain the difference between hands and tools is the fact that hands and tools putatively have a different elongation when compared to animals (i.e., tools are typical elongated objects). For instance, Gurariy et al.^57^ demonstrated that elongation and toolness are independently coded within the EEG signal, showing that decoding classification is enhanced for tools, specifically when they exhibit elongation. In a related fMRI study, Chen et al.^58^ have suggested that toolness and elongation coexist and independently influence the activation and connectivity of the tool network.

Later in the processing of these categories—around ~ 252-328 ms after stimulus onset—we showed that hands were more dissimilar from animals than tools were from animals—i.e., accuracy for hands (vs. animals) was higher than tools (vs. animals). During this time window, this heightened discriminability between hands and animals could be related to later stages associated with semantic processing. In fact, this time window follows categorical processing (e.g., N1, N2; see^59^) and precedes more integrative and conceptual components (e.g., P300, N400; see^60^). It may also be linked to differential processing associated with hands and animals in what respects biological motion and social representations that occur during hand processing. The N240 component, for example, has been shown to originate in the STS^61^, a region involved in social processing of stimuli, as well as facial and body expression^62–65^. Interestingly, we previously found that STS plays an important role during hand processing in the overlap regions^18^.

Another important test to the neural and cognitive overlap between hand and tool processing, is whether processing at a particular time point for one of the categories can be generalized to the processing of the other category. To test this, we employed a cross-classification approach, where we tested the ability of a classifier trained on classifying tools vs animals (or hands vs. animals) to classify hands as tools (or tools as hands). We found that classifiers trained on classifying tools vs. animals were significantly biased to classify hands as tools (more so than as animals) around 260–320 ms and around 376–500 ms post-stimulus onset. These results suggest that category-specific information from tools is represented in a way that is sufficiently similar to how hand-related information is coded around these time intervals in order to allow for generalization from tool processing to hand processing.

A possible interpretation of this result is that as a consequence of the processes at play (potentially automatically) when seeing a tool^66,67^, there is information about a tool—its graspable status and its associated motor program—that is minimally shared (or at least related) with hand-specific computations in order to implement those action programs. These kinds of interactions could potentially be responsible for the generalization effects seen here. Interestingly, processing of certain grasp-related information seems to happen within these time windows: De Sanctis and colleagues^60^ measured the EEG correlates during grasping movements and found grasp-specific activation peaking at 300 ms over parietal regions that continued to the central and frontal electrodes at around 400 ms.

This effect could also be related to computations happening within the ventro-dorsal pathway—a critical pathway for tool use^68^. For instance, in a study combining fMRI and EEG^69^, identified cortical regions, as well as temporal dynamics, associated with correct and incorrect use of tools. Correct use of tools led to occipitoparietal and frontal activations typically associated with the tool network. Additionally, EEG analysis showed that correct tool use activation occurred between 300 and 400 ms. In a MEG study, Suzuki and colleagues^70^ investigated the neural responses to visible and invisible images of tools. They found a strong neural response to visible images of tools in left parietal regions at 400 ms. These results suggest that the ability for a classifier trained on categorizing tools vs. animals to classify hands at that time window is dependent on action-related computations that connect hands and tools.

This result may also provide insight on the spatial overlap shown in fMRI for the processing of hands and tools—perhaps it partially occurs during the time window when these categories share action-related information. This may be particularly true for the spatial overlap in neural responses for hands and tools in the IPL because of the role of this area in accessing manipulation knowledge^38,71–74^.

Interestingly, we also showed that training on tools vs. animals around ~ 300 ms and later around ~ 400 ms provides enough information to classify hands as tools both earlier (~ 176 ms for training ~ 300 ms, and from ~ 260 ms for training ~ 400 ms) and later (until 500 ms) than those time points. This suggests the kind of tool-related action processing on which hand (action-related) processing is contingent upon occurs at ~ 300 ms and ~ 400 ms. Importantly, it has been shown that tool-specific affordances are coded around these temporal windows: for instance, in an EEG study, Proverbio and colleagues^45^ compared tools and non-tools and found that action affordance is computed at ~ 250 ms. Our data then suggest that some properties of hands could be distinguished using the action affordance at ~ 250 ms, but perhaps not the most central ones, as the contingency occurs beyond 250 ms.

Finally, when testing the ability of a classifier trained on classifying hands vs. animals to classify tools as hands, we found that this is only possible around ~ 150 ms post-stimulus onset. Nevertheless, the generalization across time extends this result for training, showing that training on hands vs. animals at different time intervals (~ 250-410 ms) allows the classification of tools as hands at ~ 150 ms. The generalization across time is again observed at a later stage: training at ~ 500 ms and testing between 400 and 500 ms. This means that the classification of hands, and especially in comparison with animals, may not lean necessarily on action-related (or other high-level) aspects, and thus, the generalization to tools may not be as clear as it was for the case of hands. The cross-classification effect that only happens at ~ 150 ms could be explained by the previously mentioned μ rhythm suppression effect^56^, suggesting that during this early time point important motor information can be extracted. The fact that the generalization only occurs again at a later stage is consistent with the hypothesis that a specific sort of action information that plays a significant role in the information transmitted between hands and tools is the grasping information that happens at around 400ms^60^. In addition, (functional) grasping information appears to be more relevant for hand processing than for tool processing areas, since prior research has demonstrated that use appropriate grasps automatically engage visual regions specialized for representing hands but not for tools^75^. This differentiation between tool and hand representations strongly suggests a processing contingency whereby action-related hand processing depends upon tool-related action processing. Lastly, while we acknowledge that no actual actions were carried out in the study and only images were viewed, it is essential to highlight that the observed results manifest at later time intervals, which cannot be attributed to low-level features. If low-level features were the sole driver, we would expect to observe results within a very early time window, typically preceding 100 ms.

An essential step in interpreting these results in a broader context is to integrate methods that offer high spatial and high temporal resolution within the same study. The intrinsic limitation of EEG's low spatial resolution presents challenges in obtaining meaningful and interpretable data at the individual electrode level. Given this limitation, and since our primary goal was to emphasize the exploration of temporal patterns, we did not intend to explore the spatial patterns of our results. Nevertheless, we believe it is pertinent to conduct a study employing fMRI-EEG fusion^76^ or source localization combined with MVPA^77^. This fMRI/EEG fusion approach, as demonstrated by Cichy and Oliva^76^, would provide us with insights into the spatial localization (fMRI) of the temporal differences (EEG) between hand and tool processing. For instance, these spatio-temporally-resolved approaches could be instrumental in determining whether regions of overlap exhibit consistent or distinct temporal dynamics. Additionally, and although it could have been important to look at cross-decoding between hand postures and tools, our current design did not allow for this analysis because we would be using only half of the data and thus this analysis would be underpowered. In a future study, we aim to address this limitation by implementing a more robust experimental design to reevaluate the potential cross-decoding effect between hand postures and tools.

In conclusion, our results show both differences and similarities between the time-course of hand and tool processing. We demonstrated that not only are the two categories processed differently over time, but that tool representations can also be informative (in specific time points) for hand processing. The fact this outcome was more narrowed in time for hand representations supports a processing contingency in which action-related hand processing is tied to the processing of action attributes during tool processing. Together these results shed new light on the respective stages of hand and tool processing and highlight how it is possible to disentangle their processing.

### Supplementary Information

Supplementary Information.

## Data Availability

The dataset utilized in the present study can be accessed on the OSF platform: https://osf.io/t4z2f/.
