# Supplementary material for: Temporal differences and commonalities between hand and tool neural processing
Source: Sci Rep. 2023 Dec 14;13:22270. doi: 10.1038/s41598-023-48180-8 (PMC10721913; doi:10.1038/s41598-023-48180-8)
Supplement: Supplementary file 1 — Supplementary Information. [file 41598_2023_48180_MOESM1_ESM.pdf]

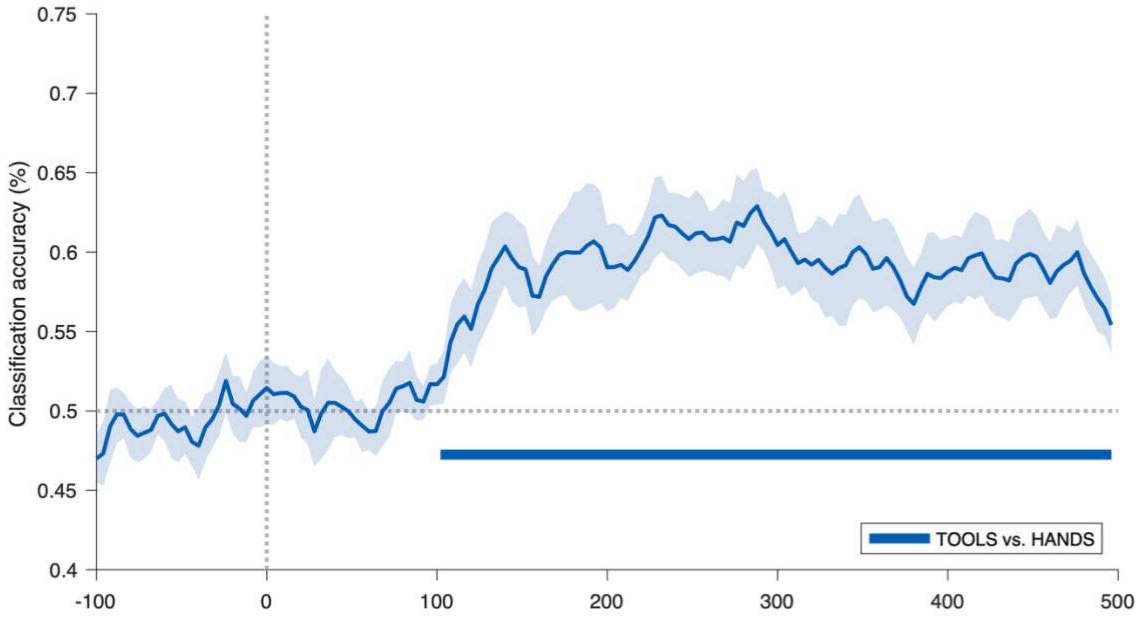

Supplementary Figure S1. Classification accuracy for tools vs. hands. The discrimination between tools and hands (blue line) was significant starting at ~104ms (early peak: t-point = 140ms, z-value = 2.33, acc = .60; mid peak: t-point = 232ms, z-value = 2.33, acc = .62; later peak: t-point = 288ms, z-value = 2.33, acc = .63).

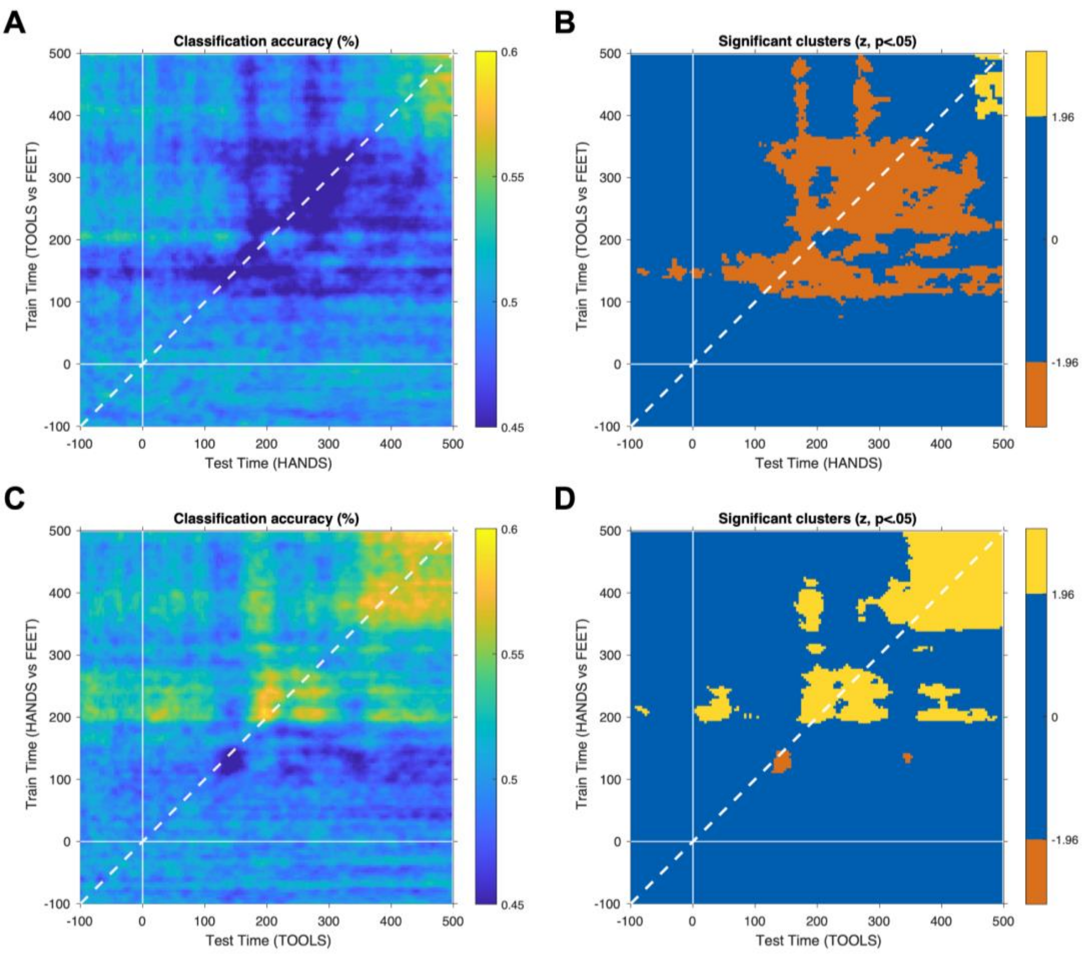

Supplementary Figure S2. Results from time generalization approach using feet as the control category. (A) Classification accuracy across time when the classifier trained on tools vs. feet and was then <u>tested on hands</u>. **(B)** The yellow color represents the significant time points when classifying hands as tools and the orange color represents classifying hands as feet. **(C)** Classification accuracy across time when the classifier trained on hands vs. feet and was then <u>tested on tools</u>. **(D)** The yellow color represents the significant time points when classifying tools as hands and the orange color represents classifying tools as feet.

The significant clusters ($|z| > 1.96$) were cluster-wise corrected for multiple comparisons using TFCE transform and maximal statistic permutation testing.
